# Protective and restorative potency of Vitamin D on persistent biochemical autistic features induced in propionic acid-intoxicated rat pups

**DOI:** 10.1186/1472-6882-14-416

**Published:** 2014-10-25

**Authors:** Hanan A Alfawaz, Ramesa Shafi Bhat, Laila Al-Ayadhi, Afaf K El-Ansary

**Affiliations:** Department of Food Science and Nutrition, College of Food and Agriculture Sciences, King Saud University, Riyadh, P.O. Box 22452, Saudi Arabia; Autism Research and Treatment Center, Department of Physiology, Faculty of Medicine King Saud University, Riyadh, Saudi Arabia; Biochemistry Department, Science College, King Saud University, P.O box 22452, Zip code 11495 Riyadh, Saudi Arabia; Prince Mutaib Chair for Biomarkers of Osteoporosis, Biochemistry Department, College of Science, King Saud University, Riyadh, 11451 Kingdom of Saudi Arabia

**Keywords:** Vitamin D, Autism, Serotonin, Glutathione-s-transferase, Interferon gamma, Comet DNA assay

## Abstract

**Background:**

Reducing exposure to toxic environmental agents is a critical area of intervention. Prenatal or postnatal exposure to certain chemicals has been documented to increase the risk of autism spectrum disorder. Propionic acid (PA) found in some foods and formed as a metabolic product of gut microbiota has been reported to mediate the effects of autism. Results from animal studies may help to identify environmental contaminants and drugs that produce or prevent neurotoxicity, and may thereby aid in the treatment of neurodevelopmental disorders such as autism. The present study investigated the protective and/or therapeutic effects of vitamin D against brain intoxication induced by propionic acid (PPA) in rats.

**Methods:**

Twenty-eight young male Western Albino rats were enrolled in the present study. They were grouped into four equal groups of 7. The control group received only phosphate buffered saline; the oral buffered PPA-treated group received a neurotoxic dose of 250 mg/kg body weight/day for 3 days; and the Vitamin D-protected group received 1000 IU/kg/day of alpha, 25-dihydroxyvitamin D (3) (1, 25-VD) for two weeks, after which the rats were injected with PPA 250 mg/Kg body weight/day for 3 days. The fourth group received PPA 250 mg/Kg body weight/day for 3 days followed by alpha, 25-dihydroxyvitamin D (3) (1, 25-VD) for two weeks (Vitamin D therapeutic effect). Vitamin D and calcium were measured in the plasma of the four studied groups. Serotonin, interferon gamma (IFN-γ), glutathione-s-transferase activity and DNA double helix breaks were assayed in the brain tissue of the rats for all groups.

**Results:**

The obtained data showed that the PPA-treated group demonstrated higher plasma vitamin D levels compared to the control rats, together with multiple signs of brain toxicity, as indicated by a depletion of serotonin (5HT), an increase in IFN-γ and inhibition of glutathione-s-transferase activity as three biomarkers of brain dysfunction. Additionally, Comet DNA assays showed remarkably higher tail length, tail DNA % damage and tail moment as a neurotoxic effect of PPA.

**Conclusions:**

Vitamin D showed a greater protective than therapeutic effect on PPA-induced neurotoxicity in rats, as there was a remarkable amelioration of the impaired biochemically measured parameters representing neurochemical, inflammation, and detoxification processes.

## Background

Autism spectrum disorder (ASD) is a group of developmental disabilities. Autism is just one disorder on the spectrum. ASD affects both the brain and body of young children. These children have atypical development regarding their socialization, communication, and behavior [1]. Despite decades of research, autism remains an idiopathic disorder in 90% of cases [1]. The most recent estimate, based on 2008 data from the Centers for Disease Control surveillance network, reveals that 1 in 88 children have autism, reflecting an almost 80% increase from 2002 data [2]. Despite thousands of studies on the etiological mechanisms of autism, the pathogenesis of these disorders remains baffling, apart from a general belief that they derive from an interaction between several genes and the environment. Among environmental factors, only some uncommon viral infections and certain drugs have been conclusively linked to autism [3].

Epidemiologists suggest that the current epidemic of autism may be caused by gestational and early childhood vitamin D deficiency [4]. A hypothesis was proposed by Cannell [5] that children with vitamin D deficient rickets have some autistic markers that seem to disappear with high-dose vitamin D treatment. Moreover, estrogen and testosterone have very different effects on calcitriol metabolism; these differences may explain the 4:1 male/female gender ratio in autism. Autism is more frequent in areas of impaired UVB diffusion and in dark-skinned persons [6, 7]. Additionally, families of higher socioeconomic status [8], as well as with a higher level of education [9] are more likely to apply sunscreen to their children, which explains the numerous studies linking higher social class with autism.

Of the neurosteroids involved in brain development, activated vitamin D (calcitriol) is extraordinarily neuroprotective by inhibiting the production of nitrous oxide, stimulating neurotropin release, reducing toxic calcium levels in the brain, reducing inflammatory cytokines [10], and increasing brain glutathione through its immunomodulating properties [11].

A placebo-controlled study of 20 autistic children over a period of three months found that multivitamins with even low doses of vitamin D (150 units or 3.75 mcg) significantly improved sleep and gastrointestinal problems [12–14]. Surprisingly, the Food and Nutrition Board (FNB) did not increase the recommended dose of vitamin D for infants, children, or young pregnant women during the decades in which sun-avoidance was routinely recommended [15]. Where there is inadequate daily sun exposure, oral doses of 1,000-2,000 IU/d are now considered routine, with much higher doses (up to 50,000 IU) for rapid repletion being considered safe [16]. Pryor found that the treatment of one autistic 26 kg boy with 3,000 IU/d of vitamin D for 3 months resulted in an increase in his 25-hydroxy vitamin D level, with concomitant improvements in behavior, learning and IQ scores. (J. Pryor, personal communication, 2008). Kalueff et al. [17] went even further, suggesting that vitamin D offers “neuroprotection, antiepileptic effects, immunomodulation, possible interplay with several brain neurotransmitter system and hormones, as well as regulation of behaviours”.

Most recently, Mostafa and Al-Ayadhi [18] reported that autistic children had significantly lower serum levels of 25-hydroxy vitamin D than healthy children. They proved that serum 25-hydroxy vitamin D had significantly negative correlations with scores on the Childhood Autism Rating Scale. Additionally, increased levels of anti-myelin-associated glycoprotein (anti-MAG) auto-antibodies were found in 70% of autistic patients and were negatively correlated with vitamin D levels. In a recent study by El-Ansary et al. [19], propionic acid was used to induce persistent autistic features in rat pups through the alteration of a panel of biomarkers comparable to those reported clinically in autistic patients and in animal models produced through interventricular infusion of PPA [20, 21].

The present study aims to ascertain the protective and therapeutic effects of vitamin D against propionic acid (PPA)-induced autistic features in animal models. Serotonin, IFγ, GST (glutathione-S-transferase), and DNA double strand breaks (Comet assay) were selected as biochemical parameters related to neurotransmission, inflammation, detoxification and DNA damage, respectively. This could highlight the role of vitamin D in the pathophysiology and treatment of autism.

## Methods

### Materials

#### Reagents

A serotonin ELISA kit, a product of Immuno-Biological Laboratories (IBL, Hamburg, Germany); an IFN*γ* ELISA kit, a product of Thermo Scientific (Rockford, IL, USA); and a glutathione-S-transferase assay kit, a product of Biovision, USA, were used in the present study. All chemicals used were of analytical grade and were products of Sigma-Aldrich, St Louis, MO, USA.

#### Equipment

An HPLC system, an ELIZA instrument, electrophoresis equipment, and a Leitz Orthoplan epifluorescence microscope (magnification 250×) equipped with an excitation filter of 515 to 560 nm and a barrier filter of 590 nm were used in the present study.

#### Animals

This is an interventional experimental animal study performed on twenty-eight male western albino rats (45–60 g) (approximately 21 days old). Rats were obtained from the animal house of the Pharmacy College, King Saud University. They were kept under standard conditions of temperature, a 12-h dark/light cycle and free access to tap water and standard laboratory chow. After one week of acclimation, the rats were divided into four groups (seven rats per group). The first group of control animals were fed with a normal diet during the experimental period; the second group of PPA-treated rats received 250 mg/Kg body weight/day for 3 days to induce autistic features; the third group consisted of the protective group, and received 1000 IU/kg/day of alpha, 25-dihydroxyvitamin D (3) (1, 25-VD) for two weeks, after which they were treated with PPA (250 mg/Kg body weight/day for 3 days). The fourth group received PPA 250 mg/Kg body weight/day for 3 days followed by alpha, 25-dihydroxyvitamin D (3) (1, 25-VD) for two weeks (Vitamin D therapeutic effect). PPA and vitamin D were given orally to rat pups using a gastric tube. All groups of rats were housed under controlled temperature conditions (21 ± 1°C) with ad libitum access to food and water. The rats were weighed daily.

#### Ethical approval

This work was approved by the Ethics Committee of the College of Science Research Center of King Saud University, Riyadh, Saudi Arabia (Approval number is 8/25/220358).

### Methods

#### Tissue preparation

At the end of the feeding trials, the rats were anesthetized with carbon dioxide and decapitated. The brain was removed from the skull and was dissected into small pieces and homogenized as a whole in 10 times w/v bi-distilled water and kept at -80°C until further use for different biochemical analyses.

#### Collection of plasma

Plasma was collected from the four investigated groups. Vitamin D and calcium were analyzed using HPLC at the facilities of the Prince Mutaib Chair for Biomarkers of Osteoporosis, King Saud University, Riyadh, KSA.

#### Assay of serotonin

Serotonin was measured using an ELISA kit from Immuno-Biological Laboratories (IBL, Hamburg, Germany). The assay procedure followed the competitive ELISA protocols, whereby competition takes place between the biotinylated and non-biotinylated antigen for a fixed number of antibody binding sites. Brain homogenate preparation (derivatization of serotonin to N-acylserotonin) was part of the sample dilution. Briefly, serotonin in samples and controls was acylated with acetic anhydride in acetone and samples, controls, and standards were applied to 96-well microtiter plates coated with goat anti-rabbit IgG. Biotinylated serotonin and rabbit antiserum to serotonin were added to each well and incubated overnight at 4°C. Para-nitrophenyl-phosphate in a diethanolamine solution was used as a substrate following the application of alkaline phosphatase-conjugated goat anti-biotin antibody. Samples were read at 405 nm on an ELISA plate reader and quantified using standards supplied by the manufacturer. The analytical sensitivity of this product is 0.014 ng/ml.

#### Assay of IFNγ

IFN*γ* was measured using an ELISA kit, a product of Thermo Scientific (Rockford, IL, USA), according to the manufacturer’s instructions. This assay employs a quantitative sandwich enzyme immunoassay technique that measures IFN*γ* in less than five hours. In this kit, a polyclonal antibody specific to human IFN*γ* is pre-coated onto a 96-well microplate with removable strips. IFN*γ* in 100 μl standards and samples was sandwiched by the immobilized antibody and a biotinylated polyclonal antibody specific to IFN*γ*, which is recognized by a streptavidin-peroxidase conjugate. All unbound material was then washed away and a peroxidase enzyme substrate was added. The color development was stopped and the intensity of the color was measured at 550 nm and subtracted from the absorbance at 450 nm. The minimum level of rat IFNγ detected by this product was less than 2 pg/ml.

#### Determination of glutathione-S-transferase activity (GST)

Ten-microliter samples were prepared in a total 50 μl volume with GST assay buffer, including a negative control with 50 μl of GST assay buffer only and a positive control (10 μl of GST positive control diluted 1:50) with 40 μl of GST assay buffer. Five microliters of glutathione (GSH) was added to each well containing the sample or control described above. Fifty microliters of 10-times diluted CDNB (1-chloro- 2, 4-dinitrobenzene) was mixed and added to all wells, including the standard. The GST-catalyzed formation of GS-DNB produces a dinitrophenyl thioether, which can be detected by a spectrophotometer at 340 nm.

#### Comet DNA assay

Brain tissue collected from the rat samples was homogenized in 0.075 M NaCl and 0.024 M ethylenediaminetetraacetic acid (EDTA) buffer, pH 7.5, at a ratio of 1 g of tissue to 1 ml of buffer, and then cooled to 4°C. Volumes of 6 μl of brain homogenate were suspended in 100 μl of 0.5% low-melting agarose (LMA) (Sigma-Aldrich, St Louis, MO, USA) and placed onto microscope slides that were cleaned and coated with 300 μl of 0.6% normal melting point (NMP) agarose beforehand. After solidification on ice for 10 minutes, the slides were covered with 0.5% low melting point (LMP) agarose. Once the agarose gel solidified, the slides were immersed for one hour in an ice-cold lysis solution, consisting of 100 mM Na_2_EDTA, 2.5 M NaCl, 10 mM Tris–HCl, and 1% sodium sarcosinate, which was adjusted to pH 10 using 1% Triton X-100 and 10% dimethyl sulfoxide (DMSO), added immediately prior to use. Before electrophoresis, the slides were removed from the lysing solution and placed for 20 minutes in a horizontal electrophoresis unit (near the anode) that was filled with an alkaline buffer to allow the unwinding of the DNA and to express alkali-labile damage. The electrophoresis alkaline solution consisted of 1 mM Na_2_EDTA and 300 mM NaOH, pH 13. After the unwinding of the DNA, electrophoresis was carried out in the freshly prepared alkaline solution for 20 minutes at 25 V (300 mA). Electrophoresis at a high pH resulted in structures resembling comets, as observed by fluorescence microscopy; the intensity of the comet tail relative to the head reflected the number of DNA breaks. Afterwards, the slides were neutralized by adding Tris buffer (pH 7.5), stained with 30 ml of ethidium bromide (Sigma-Aldrich, St Louis, MO, USA) (20 mg/L), and then covered and stored in sealed boxes at 4°C for further analysis.

All preparation steps were performed under dimmed light to prevent additional DNA damage. Images of 100 randomly selected cells (50 counts on each duplicate slide) were analyzed for each sample. For each group, a total of 500 cells were analyzed under a Leitz Orthoplan epifluorescence microscope (magnification 250×) equipped with an excitation filter of 515 to 560 nm and a barrier filter of 590 nm. The microscope was connected through a camera to a computer-based image analysis system (Comet Assay IV software, Perspective Instruments).

Comets were randomly captured at a constant depth of the gel, avoiding the edges of the gel, occasional dead cells, and superimposed comets. DNA damage was measured as tail length (TL = distance of DNA migration from the center of the body of the nuclear core) and DNA tail intensity (TI = % of genomic DNA that migrated during the electrophoresis from the nuclear core to the tail). By presenting all three parameters together, more information could be obtained on the extent of the DNA damage.

#### Statistical analysis

The data were analyzed using the Statistical Package for the Social Sciences (SPSS, Chicago, IL, USA). The results were expressed as the mean ± standard error of the mean (SEM). All statistical comparisons between the control and PA-treated rat groups were performed using the one-way analysis of variance (ANOVA) test complemented with the Dunnett test for multiple comparisons. Significance was assigned at the level of *P* <0.05. Receiver operating characteristics curve (ROC) analysis was performed. Area under the curve (AUC), cut-off values, and degree of specificity and sensitivity were calculated. Pearson correlations were calculated.

## Results

**Figure 1:**
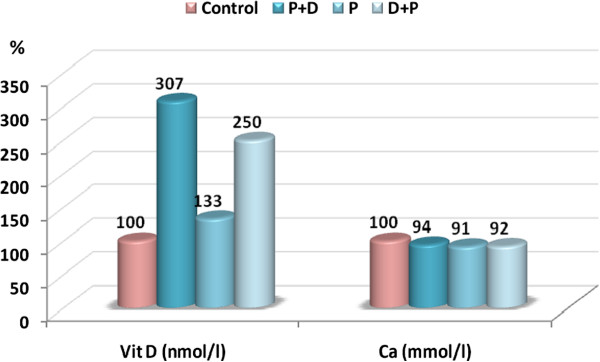
**Percentage change in vitamin D and calcium in the plasma of the PPA, protected (PPA + D), and therapeutic (D + PPA) groups compared to the control.**

**Figure 2:**
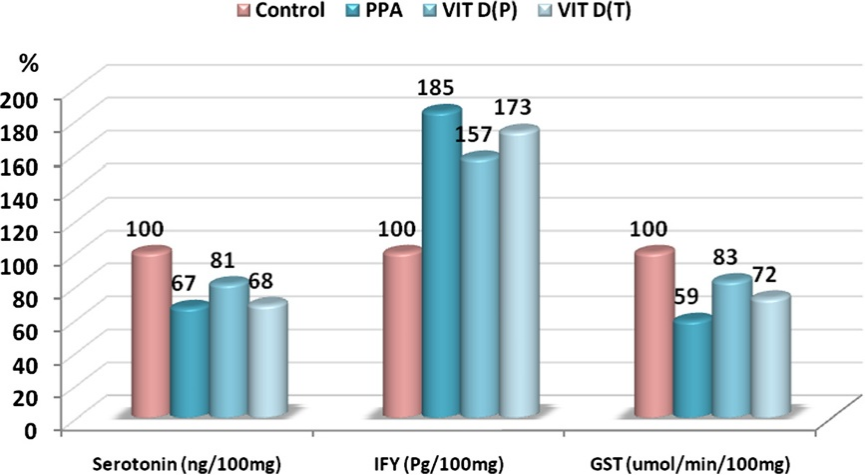
**Percentage change in serotonin, IFγ and GST in the three treated groups compared to the control.**

**Figure 3:**
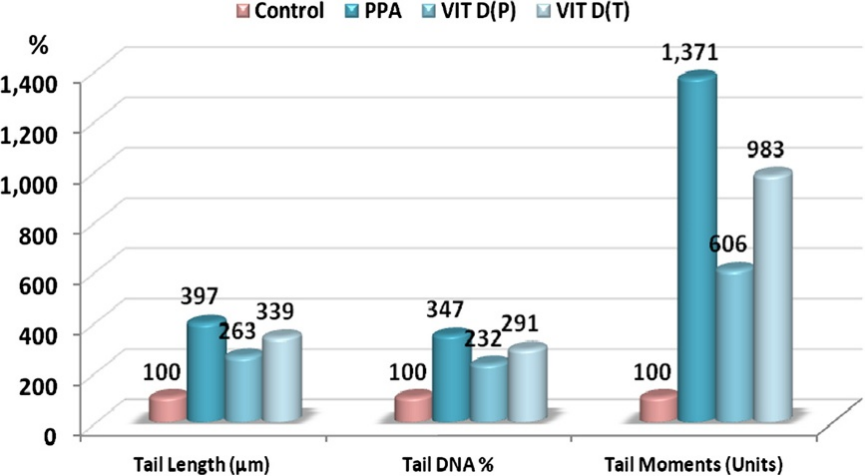
**Percentage change in tail length, tail DNA% and tail moment in the three treated groups compared to the control.**

**Figure 4:**
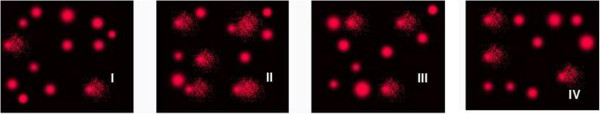
**Photograph showing comet tailing in the PPA-treated group (II) together with the protective and therapeutic effects of vitamin D (III &IV) in rat brains, compared to control (I).**

**Figure 5:**
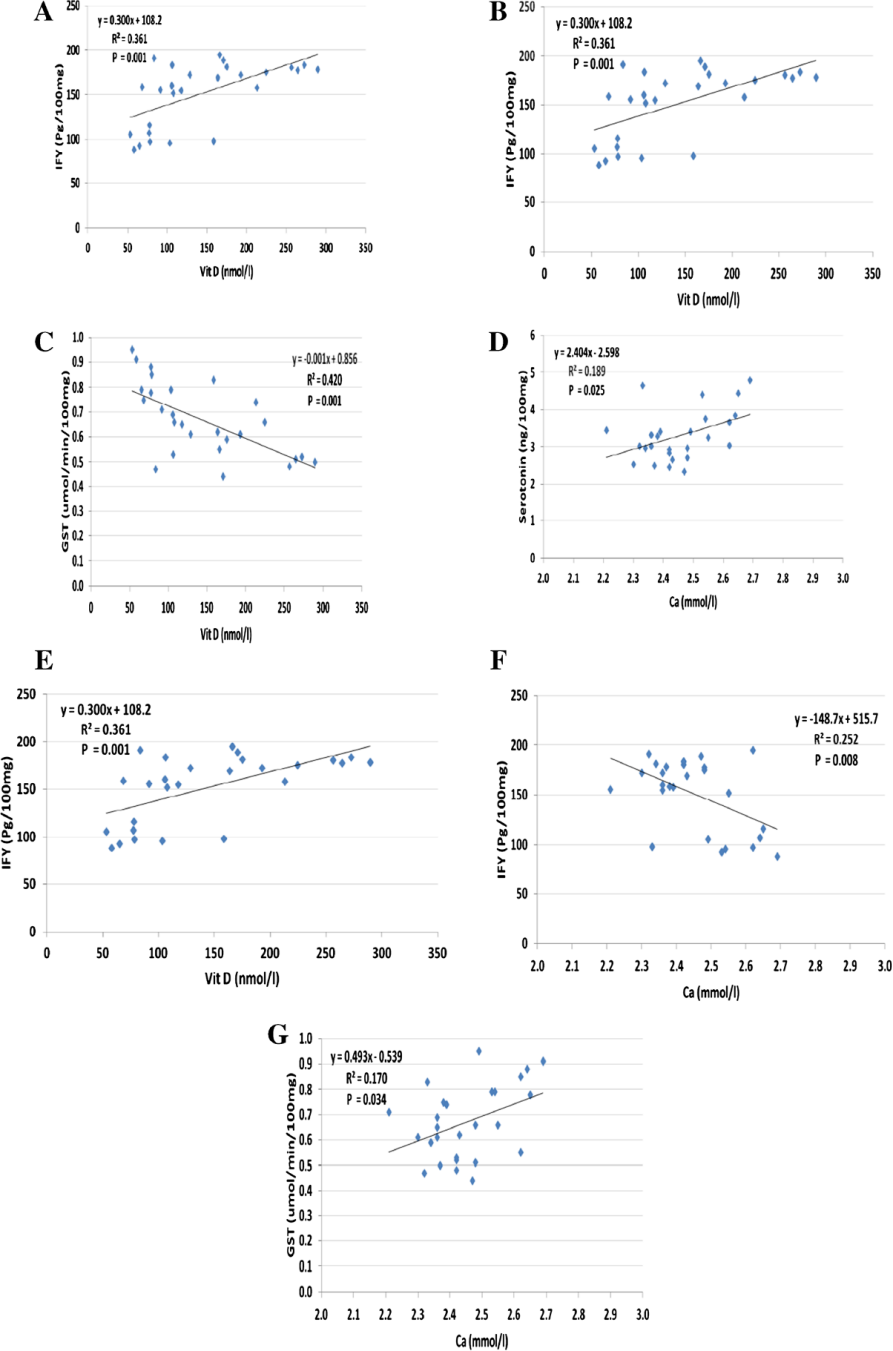
**Comet assay performed. (A-G)**: Pearson correlations between plasmatic levels of vitamin D, calcium and different measured brain parameters.

Data are presented in Tables 1, 2, and 3 together with Figures 1, 2, 3, 4, and 5. Table 1 lists the plasma levels of vitamin D and calcium presented as the mean ± S.D for the four studied groups. It can be easily noticed that vitamin D was higher in the PPA-treated group compared to the control and, as expected, was remarkably and significantly elevated in the protected and therapeutically treated groups compared either to the control or PPA-treated groups. In contrast, calcium shows non-significant variation between the four studied groups. Figure 1 presents the percentage change of vitamin D and calcium in the treated groups relative to the control (presented as 100%). It can be easily observed that a 33%, 207% and 150% increase in vitamin D levels was recorded for PPA-treated, vitamin D- protected and vitamin D-treated groups, respectively. Figure 1 also demonstrates the non-significant change in calcium levels.

**Table 1:** **Plasma levels of Vitamin D (nmol/l) and Ca (mmol/l) in the four studied groups**

Parameters	Groups	Min.	Max.	Mean ± S.D.	P value
Vit D (nmol/l)	Control	52.98	103.56	73.36 ± 16.78	0.001
	PPA + D	158.80	289.60	225.50 ± 57.42^a^	
	PPA	68.30	117.60	97.28 ± 17.03	
	D + PPA	128.68	224.40	183.08 ± 34.92^a^	
Ca^+2^ (mmol/l)	Control	2.49	2.69	2.59 ± 0.08	NS
	PPA + D	2.33	2.62	2.44 ± 0.09	
	PPA	2.21	2.55	2.37 ± 0.10	
	D + PPA	2.30	2.48	2.38 ± 0.06	

• Table 1 describes the one-way ANOVA test between the control, P + D, P and D + P groups in Vit D (nmol/l) and Ca (mmol/l) groups and the Dunnett test for multiple comparisons.

Significant differences between the three groups are illustrated as superscript letters when P <0.05.

**Table 2:** **Serotonin (ng/100 mg), IFγ (Pg/100 mg) and GST (μmol/min/100 mg) groups**

Parameters	Groups	Min.	Max.	Percent change	Mean ± S.D.	P value
Serotonin (ng/100 mg)	Control	3.41	4.80	100.00	4.12 ± 0.51	0.001
	PPA	2.32	3.03	66.86	2.76 ± 0.29^a^	
	VIT D(P)	3.25	3.45	80.97	3.34 ± 0.07^a^	
	VIT D(T)	2.52	3.06	68.41	2.82 ± 0.22^a^	
IFγ (Pg/100 mg)	Control	88.27	115.64	100.00	99.91 ± 8.84	0.001
	PPA	177.34	194.59	184.88	184.72 ± 6.24^a^	
	VIT D(P)	152.05	160.01	156.62	156.49 ± 2.95^a^	
	VIT D(T)	165.48	181.05	172.84	172.68 ± 5.28^a^	
GST (μmol/min/100 mg)	Control	0.78	0.95	100.00	0.85 ± 0.06	0.001
	PPA	0.44	0.55	59.00	0.50 ± 0.04^a^	
	VIT D(P)	0.65	0.75	82.77	0.70 ± 0.04^a^	
	VIT D(T)	0.59	0.66	72.35	0.61 ± 0.02^a^	

• Table 2 describes the One-way ANOVA test between the control, PPA and vitamin D (P&T) groups in serotonin (ng/100 mg), IFγ (Pg/100 mg) and GST (μmol/min/100 mg) groups and the Dunnett test for multiple comparisons.

Significant differences between the three groups are illustrated as superscript letters when P <0.05.

**Table 3:** **Tail Length (μm), Tail DNA% and Tail Moments (Units) groups**

Parameters	Groups	Min.	Max.	Percent change	Mean ± S.D.	P value
Tail Length (μm)	Control	1.06	1.42	100.00	1.25 ± 0.16	0.001
	PPA	4.67	5.34	396.92	4.96 ± 0.28^a^	
	VIT D(P)	2.97	3.66	262.73	3.28 ± 0.33^a^	
	VIT D(T)	3.96	4.52	339.37	4.24 ± 0.23^a^	
Tail DNA%	Control	1.22	1.62	100.00	1.40 ± 0.17	0.001
	PPA	4.51	5.15	347.44	4.85 ± 0.27^a^	
	VIT D(P)	3.11	3.41	232.49	3.25 ± 0.12^a^	
	VIT D(T)	3.82	4.34	291.35	4.07 ± 0.23^a^	
Tail Moments (Units)	Control	1.42	2.15	100.00	1.76 ± 0.37	0.001
	PPA	21.96	25.78	1370.59	24.07 ± 1.87^a^	
	VIT D(P)	9.91	11.84	605.97	10.64 ± 0.87^a^	
	VIT D(T)	15.12	18.51	983.37	17.27 ± 1.49^a^	

• Table 3 describes the one-way ANOVA test between the control, PPA and OMEGA groups in Tail Length (μm), Tail DNA% and Tail Moments (Units) groups and the Dunnett test for multiple comparisons.

Significant differences between the three groups are illustrated as superscript letters when P <0.05.

Table 2 and Figure 2 demonstrate a significant impairment in the production of serotonin, IFγ and GST induced by PPA, together with the protective and restorative effects of vitamin D. Vitamin D shows more protective than therapeutic effects, but there was still a significant difference when compared to the control group. This is shown graphically in Figure 2 as a lower % change compared to the control than that seen after PPA-treatment.

Table 3 and Figure 3 demonstrate PPA-induced DNA damage in the brains of treated rats. The evidence for DNA damage is the significant increase in the comet parameters, presented as tail length (μm), tail DNA (%) and tail moment (arbitrary units). Table 3 also demonstrates the potency of vitamin D in protecting against and treating PPA neurotoxicity, and how it ameliorates the DNA-damaging effects of PPA. This amelioration was observed as a significant decrease in PPA-induced DNA damage. ROC analysis showed satisfactory values of area under the curve, sensitivity and specificity.

## Discussion

Nutritional factors play an important role in promoting good health, and a preponderance of evidence has linked nutritional deficiencies to an exacerbation of cognitive deterioration. Recently, vitamin D has come under investigation for its role in cognitive preservation. While a role for vitamin D in tissue growth and bone metabolism is well established, the presence of the vitamin D receptor and enzymes involved in the hydroxylation of vitamin D (25-OHase and 1,a-OHase) in the brain implies a role for this hormone in cognitive function and dementia [22–24].

Circulating 1,25(OH)2D enters cells by passive diffusion and binds to a nuclear vitamin D receptor (VDR), causing a conformational change that allows the VDR to dimerize with the retinoid X receptor [25]. VDRs and/or 1α-hydroxylase have been identified in a wide range of cells, including brain cells. While the importance of vitamin D levels for normal bone formation, parathyroid function and intestinal calcium absorption are well documented, the levels needed for optimal brain development and function are unknown [26–29].

Table 1 demonstrates a higher level of vitamin D in the plasma of PPA-treated rats compared to the control. Because the neurotoxicity of both intraventricularly and orally administered PPA was recently ascertained [19, 20], higher plasmatic vitamin D could reflect a lower brain concentration, which could be due to an abnormally low number of VDRs in the brain of PPA-treated rats. While some studies reported no observed impairments in working memory or anxiety in the VDR-KO model mouse [30], others showed anxiety-like behavior and behavioral impairment [31, 32].

Table 2 demonstrates that vitamin D shows more protective than therapeutic effects against PPA neurotoxicity. The remarkable protective effects of vitamin D against PPA’s negative effect on serotonin levels reported in the present study are in concurrence with the previous work of Cass et al. [33], which reported that calcitriol was effective in protecting against methamphetamine (METH)-induced reductions in striatal and nucleus accumbens levels of dopamine and serotonin (5HT) in male rats. Moreover, the suggested lower number of VDRs as a neurotoxic feature of PPA could be supported by considering the protective effect of vitamin D via upregulation of VDR mRNA 12–24 hr after brief glutamate exposure in cultured neurons. This could suggest that vitamin D3 may play a role in mechanisms relevant to protection against PPA neurotoxicity through up-regulation of VDR expression in the brains of treated rats.

Regarding the anti-inflammatory effect of vitamin D, it can easily be observed from Table 2 that vitamin D could lower the percentage increase in IFN-γ from 85% in PPA-treated unprotected rats to 57% in the vitamin D-protected group. In contrast, vitamin D shows a very low therapeutic potency. These results could confirm the anti-inflammatory effects of vitamin D through the suppression of pro-inflammatory cytokines and the inhibition of NF-κB signaling [10].

The remarkable inhibition of GST reported in the present study could easily be related to the oxidative effect of PPA previously reported as increased lipid peroxides, depleted glutathione and less active glutathione peroxidase [19].

Previous studies have shown that vitamin D could protect the structure and integrity of neurons through detoxification mechanisms and neurotrophin synthesis [34–38]. Similar to the benefits of traditional antioxidant nutrients, 1,25(OH)2D3 inhibits inducible nitric oxide synthase (iNOS) [39], an enzyme that is upregulated during ischemic events and in patients with Alzheimer’s, Parkinson’s disease and autism [40]. 1, 25(OH) 2D3 also enhances innate antioxidant pathways, upregulates gamma glutamyl transpeptidase [41] and subsequently increases glutathione. Glutathione is an innate antioxidant that protects oligodendrocytes and the integrity of the nerve conduction pathway critical to mental processing. These earlier findings could be supported by the present study, in that vitamin D attenuates the oxidative effect of PPA and enhances the detoxification mechanism through the activation of GST.

Table 3 and Figure 4 show the remarkable DNA double strand breaks with PPA, together with the potent protective and mild therapeutic effects of vitamin D supplementation. Vitamin D given before oral administration of PPA was effective at ameliorating its neurotoxic effect. Tail length was reduced from 4.95 μm to 3.28 μm (i.e., from a 397 to a 263% increase compared to the control), and % DNA damage was reduced to 3.25 compared to a value of 4.85 in PPA-treated rats. The most remarkable protective effect of vitamin D was clearly seen in tail moment, which was reduced to a value of 10.64 compared to 24.07 in PPA-treated rats (reduction in the % increase value from 1371% to 606% in vitamin-D-protected rats). In contrast, the therapeutic effect of vitamin D supplementation was remarkably less compared to its protective effect. The DNA damaging effect reported in the present study could be easily related to the previously suggested loss of VDRs with PPA treatment. This is because the elevation in the levels of 8-hydroxy-2-deoxyguanosine (8-OHdG), a marker of oxidative DNA damage, corresponded to a complete loss of VDR expression [42].

Because VDR expression is dependent on 1,25OHD availability, its loss was taken to suggest a possible role for 1,25OHD in protecting cells against hyper-proliferation and oxidative DNA damage [43]. Vitamin D-treated rats have shown a significant reduction in damage due to oxidative stress and a corresponding increase in bone density [44]. Further evidence to support the potential for vitamin D to resolve oxidative damage may be found in a randomized clinical trial in patients with colorectal adenoma where a 25% reduction in 8-OHdG following vitamin D supplementation was reported [45]. Lipid peroxidation is another marker for oxidative damage and is indicated by the formation of malondialdehyde, which plays an important role in carcinogenesis [46]. Treatment of rats with calcitriol increased the expression of VDR and greatly reduced levels of malondialdehyde, further supporting the role of high levels of calcitriol in protecting DNA against oxidative damage. All these studies could be used to support our suggestion of VDR loss as a PPA-oxidative-stress-related neurotoxicity event, and of the protective effect of vitamin D supplementation against the etiology of autistic features recently reported as a persistent neurotoxic effect of PPA.

ROC analysis shows satisfactory values for area under the curve, sensitivity and specificity. This could confirm that the measured parameters could be used as biomarkers to study either PPA neurotoxicity or vitamin D protective and/or therapeutic effects. These suggestions could be supported through the positive and negative correlations shown in Figure 4 (A-G), which help to suggest the possibility of using vitamin D supplementation during pregnancy to protect against the development of autistic features after birth.

## Conclusion

Based on the anti-inflammatory, antioxidant, and DNA repair actions of vitamin D reported in the present study, vitamin D could be used as a supplement in patients with autism to ameliorate the symptoms related to these pathways. As maternal vitamin D deficiency may predispose children to autism, a high consumption of vitamin D-rich seafood, and an avoidance of the use of sunblock during pregnancy could be suggested as an autism prevention strategy.
